# An observational time-series study on the behavioral effects of adjunctive artisanal cannabidiol use by adults with treatment resistant epilepsies

**DOI:** 10.1186/s12883-024-03646-8

**Published:** 2024-04-26

**Authors:** Barbara A. Brett, Matthieu Conroy, Hardik Doshi, Matthew X. Lowe, Sasha Kalcheff-Korn, Heather Jackson

**Affiliations:** 1 Department of Psychology, Colorado State University-Pueblo, 2200 Bonforte Blvd., Pueblo, CO 81001 USA; 2 US Army Medical Research Institute of Chemical Defense, Aberdeen, MD USA; 3 Cape Fear Valley Medical Center, Fayetteville, NC USA; 4 Realm of Caring Foundation, Colorado Springs, CO USA

**Keywords:** CBD, TRE, QoL, Anxiety, Depression

## Abstract

**Background:**

For approximately 30% of people with epilepsy, seizures are not well-controlled by anti-seizure medication (ASM). This condition, called treatment resistant epilepsy (TRE), is associated with increased morbidity and mortality, and substantially impacts the quality of life of both the individual and their family. Non-responsiveness to ASMs leads many people with TRE to seek alternative therapies, such as cannabinoid-based medication, particularly cannabidiol (CBD), with or without medical or professional advice. This is due in part to widespread reporting in the media about the benefits of CBD for seizures in some forms of epilepsy.

**Methods:**

Adults with TRE, opting to add CBD to their existing treatment regime, completed this prospective, observational, longitudinal, quasi-experimental, time-series study. We hypothesized that adjunctive CBD use would positively impact participants’ quality of life and psychological well-being in comparison to a baseline period without CBD use. Participants were followed for a period of approximately six months – for approximately one month of baseline prior to the initiation of CBD use and approximately five months after the initiation of CBD use. Participants provided urine samples and completed behavioral questionnaires that assessed quality of life, anxiety/depression, and adverse events during baseline and at two times during CBD use.

**Results:**

Complete case analyses (*n* = 10) showed a statistically significant improvement in quality of life, a statistically significant decrease in anxiety symptoms, and a statistically significant decrease in the experience of adverse events over time (*p* < 0.05). Improvements noted in the experience of depression symptoms did not reach statistical significance. Urinalysis revealed the majority of participants had no CBD/metabolites in their system at the beginning of the study, and confirmed the presence of CBD/metabolites in participants’ urine after CBD was added to their treatment regime. Analysis of missing data using multiple imputation supported the findings of the complete case analysis.

**Interpretation:**

For a small group of individuals with TRE of varying etiologies, adjunctive use of artisanal CBD was associated with improvements in the behavioral and psychological symptoms of TRE, as well as improved medication tolerability.

## Introduction

### Background

#### Epilepsy and anti-seizure medication

Epilepsy is a heterogeneous neurological condition characterized by recurrent seizures [1]. For approximately 70% of people with epilepsy, seizures are well-controlled by one or more of the ~ 30 anti-seizure medications (ASMs) currently available [2, 3].

For approximately 30% of people with epilepsy, seizures are not well-controlled by existing ASMs [4–6]. This condition, called treatment resistant epilepsy (TRE), is associated with severe morbidity, as demonstrated by health, economic, and psychosocial problems, such as increased risk of injury, employment discrimination, and difficulties with social interactions [7–9]. Not only does TRE have a negative impact on patients’ psychological well-being life, but also their quality of life.

Although ASMs effectively decrease seizure frequency in many people, they are associated with significant, adverse central nervous system effects, such as psychological and behavioral effects, cognitive effects, and memory problems [10–12]. Adverse effects of ASMs are a leading cause of epilepsy treatment failure [13]. Identifying additional treatment options for people with TRE is a high priority of researchers.

#### Objectives

The primary goal of this study was to determine if independent adjunctive use of artisanal CBD was beneficial for adults with TRE. We hypothesized that participants treating themselves with CBD would report an improvement in quality of life and anxiety/depression symptoms. We also hypothesized that CBD use would be associated with limited adverse effects.

## Methods

### Study design

This was a prospective, observational, longitudinal, quasi-experimental, time-series study examining a small cohort of adults with TRE opting to use CBD as an adjunctive treatment. All procedures were approved by the Colorado State University-Pueblo Institutional Review Board for human subjects research. All participants provided voluntary informed consent. This study is reported following STROBE guidelines [ref: https://www.strobe-statement.org/].

**Fig. 1:**
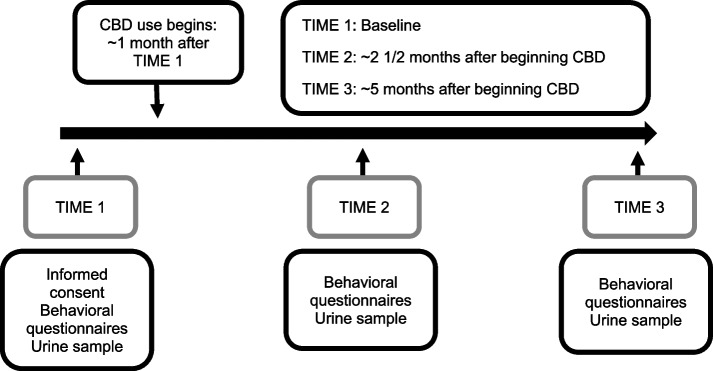
Timeline; Legend: The horizontal arrow in the center of the figure represents the six month time course of the study. Below the arrow, the three time points for data collection are displayed in boxes: TIME 1, TIME 2, TIME 3. Above the arrow, the timing of the initiation of CBD is displayed along with the ~ timing of data collection relative to the initiation of CBD use. The information collected at each time point is indicated in the lowest boxes

Participants were followed for a period of approximately six months. Participation began approximately 30 days prior to the initiation of daily CBD use and continued for approximately five months after. This time-frame was selected so that a pre-CBD baseline could be established and the presence of cannabinoids/metabolites and any sustained effects on behavior could be tracked over an extended period of time, at two time points after the initiation of CBD use. A timeline for the study is provided in Fig. 1.

### Setting

A community sample of adults with TRE was recruited to participate in this study either face-to-face or remotely between the summers of 2017 and 2019. Participants were recruited via advertisement on the Realm of Caring website, an advertisement in the University Email Digest, communication with the Epilepsy Foundation of Colorado, communication with local neurologists, announcements at conferences, and word of mouth. Researchers collected data from face-to-face participants in their home. Remote participants had study kits mailed to them and surveys were administered over the phone. In addition to following up with participants at TIME 2 and TIME 3, researchers maintained regular phone and/or email contact to address any concerns and answer any questions.

### Participants

Inclusion criteria were: male or female; 18 years of age or older; resident of Colorado or state with cannabinoids legalized for epilepsy; documentation of a diagnosis of TRE as evidenced by medical records, and/or the following clinical feature—failure to control seizures after trial of two anticonvulsant medications at therapeutic levels; verbal report of baseline seizure frequency of at least 4 seizures/28 days; and, 1– 5 antiepileptic medications at stable doses for 1 month prior to enrollment. Exclusion criteria were: verbal report of cannabinoid use within the last 30 days; and, epilepsies associated with neurodegenerative diseases and/or inborn errors of metabolism.

The inclusion criteria “resident of Colorado or a state with cannabinoids legalized for epilepsy” was used in order to legally protect the participants. The time frame during which recruitment for this study took place was a period when cannabinoid products such as CBD oil were widely available online, for example, but not necessarily fully legalized in a specific state. The inclusion criteria related to seizure frequency and antiepileptic medication were based on epileptologists’ recommendations.

### Variables

#### Behavioral questionnaires

##### The Quality of Life in Epilepsy 31-P [14, 15]

The Patient-Weighted Quality of Life in Epilepsy Inventory (QOLIE-31-P) is version 2 of the original QOLIE-31. This instrument included seven multi-item subscales that assessed: emotional well-being, social functioning, energy/fatigue, cognitive functioning, seizure worry, medication effects, and overall quality of life. The QOLIE-31-P added one new item to each subscale asking about distress, defined as bothersomeness for the respondent. Items were measured on 4- to 6- point Likert scales, with a maximum total score of 100; range = 0—100. Higher values indicated better quality of life. The validity and reliability of the original QOLIE-31were demonstrated [16]. No information about the reliability and validity of the QOLIE-31-P was found. The primary outcome measure for this study was the variable “QOLIE-31-P TOTAL SCORE”. The TOTAL SCORE data was analyzed using “Distress” as a separate subscale.

##### The Hospital Anxiety and Depression Scale (HADS [17];)

The HADS is a self-administered scale used to assess the presence and severity of anxiety and depression symptoms. The HADS consisted of 14 items that were scored on a 4-point severity scale ranging from 0 to 3, with higher scores indicating greater severity. There were two subscales (anxiety and depression), with seven items related to anxiety and seven items related to depression. Subscale scores ranged from 0 to 21. The variable “HADS” was a main outcome measure for the study. The HADS authors suggested that a score of 0 to 7 for either the anxiety or depression subscale may be regarded as being in the normal range, a score of 8 to 10 suggests the presence of the respective state, and a score of 11 or higher indicates the probable presence of clinically significant anxiety or depression. The HADS depression subscale is recognized as a valid and reliable measure of depressive symptoms in patients with epilepsy [17]. No information about the validity of the HADS anxiety sub-scale in patients with epilepsy was identified.

##### The Liverpool Adverse Events Profile (LAEP [18])

The LAEP is a 19-item self-report questionnaire that is typically used to screen for adverse effects of anti-seizure medication. It’s scored on a four-point Likert scale. Scores range from 19 – 76; higher scores indicated more side effects. The validity and reliability of the LAEP have been verified [18–20]. The variable “LAEP GLOBAL SCORE” was also an outcome measure for the study.

#### Cannabidiol use

Because this was an observational study and not a clinical trial, no CBD or cannabinoids were provided to the participants by the researchers. Products were obtained by families following state regulations. Participants were strongly encouraged to discuss participation in this study with their physician, and they were informed about potential drug interactions between CBD and ASMs [21]. Participants planning to add CBD to their treatment regime were encouraged to seek guidance about cannabinoid use from the Realm of Caring Foundation. Realm of Caring Foundation is a 501(c)(3)-non-profit organization that provides support services to people interested in the medicinal use of cannabinoids. Six of 10 participants reported using Charlotte’s Web Advanced Formula Hemp Oil Extract-only on a daily basis, beginning at a dose of approximately 0.25 mL 2x/day (= approximately 25 mg cannabinoids/CBD/day), and increasing over time, depending on their experience of benefits vs. side effects. One participant was using Epidiolex (10 mL/day). Three participants reported using a different CBD product or a variety of products including CBD.

#### Cannabinoid analysis

Urine samples were packed in ice and shipped overnight to the company iC42. Participants’ urine was batch processed for the presence of cannabinoids at the end of the study. The purpose of testing participants’ urine was to collect preliminary information about the levels of cannabinoids and metabolites present in participants’ urine over the course of the study. The assay (#2) developed by iC42 simultaneously tested for the following: Δ9-tetrahydrocannabinol (THC), 11-hydroxy-Δ9-tetrahydrocannabinol (11OH-THC), 11-nor-Δ9-tetrahydrocannabinol-9-carboxylic acid (THC-COOH), 11-nor-Δ9-tetrahydrocannabinol-9-carboxylic acid glucuronide (THC-Gluc), Cannabidiol (CBD), 6a-hydroxy cannabidiol (6a-OH-CBD), 6b-hydroxy cannabidiol (6b-OH-CBD), 7-hydroxy cannabidiol (7OH-CBD), 7-cannabidiol-9-carboxylic acid (7-CBD-COOH), Cannabidiol-9-carboxylic acid glucuronide (CBD-Gluc), Cannabichromene (CBC), Cannabinol (CBN), Cannabigerol (CBG), Cannabidivarin (CBDV), and Δ9-tetrahydrocannabivarin (THCV). See Anderson [22] for details about the LC–MS-MS methodology used to process the samples.

#### Study size

An a priori power analysis was conducted to determine the minimum sample size required to test the study hypotheses. Results indicated the minimum sample size to achieve 85% power to detect an estimated large effect (d ≥ 0.8) at a significance criterion of α = 0.05, was 34, for a within-subjects repeated measures ANOVA. Despite our best recruitment efforts, we were only able to enroll 21 participants and ten participants completed the study. This means the study was underpowered.

#### Bias

In order to address potential bias in the complete case analysis, a missing data analysis was subsequently performed on data from participants who dropped out of the study using multiple imputation (MI) (See Missing data analysis).

#### Statistical methods

This study used a within-subjects research design. Repeated measures ANOVAs were run using IBM SPSS Statistics 29 to analyze behavioral questionnaire data that was collected at three different time points (TIME 1 (baseline), TIME 2, and TIME 3). For each a priori analysis, the *p* value was set to *p* = 0.05 and confidence intervals were 95%. Effect sizes (Partial ETA) were reported. Bonferroni corrections were applied to pairwise and post hoc tests to correct for the inflation of Type 1 error. Greenhouse–Geisser corrections were utilized for violations of sphericity.

#### Missing data analysis

Missing data analysis was performed using data from 7/11 dropouts for which we had TIME 1 data as opposed to no data. The method chosen for handling missing data was multiple imputation (MI) [23, 24] The reason multiple imputation was used is because the drop-out rate in this study was high as was the percentage of data values missing for each variable, possibly leading to misleading conclusions related to our complete case analysis. MI has the potential to improve the validity of medical research (Sterne, et al., 2009), and compared to other simple forms of imputation, such as Last Observation Carried Forward (LOCF), MI does a better job of preserving variability in the imputed dataset. Using MI provided for an *n* = 17 in supplementary analyses, as opposed to the original *n* = 10 in the complete case analysis. MI was carried out in SPSS 29 (IBM). The number of imputations was set to 5. The method of analysis chosen was “monotone”. The model type was linear regression. Repeated measures ANOVAs were used to analyze data from the imputed datasets for comparison with the results from the complete case analyses.

## Results

### Participants

Eighty-one people contacted the PI about participation in this study. Thirty-five people were screened, and 21 people were determined to be eligible to participate in this study (response rate = 60.0%). Of the 14 individuals who were deemed ineligible to participate, reasons for ineligibility included: too few seizures (6/13; 42.9%); current use of CBD/cannabinoids (4/13; 35.7%); and, miscellaneous (3/14; 21.4%), such as wanting to enroll to get off of ASMs.

Of the twenty-one participants enrolled in the study, ten participants completed it. Due to their medical condition(s), the lives of individuals and families dealing with TRE are extremely stressful and difficult. Four individuals unenrolled after consenting to participate and receiving the study material but prior to providing Time 1 questionnaire data. One of these individuals was living in assisted living and their request to participate was denied. Three of these individuals and their families expressed feeling overwhelmed by adding more responsibility to their already challenging lives, despite their desire to participate in research. Four participants (M = 1, *F* = 3) who unenrolled after TIME 1 expressed similar concerns. Reasons for withdrawal of the remaining 3 participants included: moving into assisted living where cannabinoids were disallowed (M = 1), repeated hospitalizations for additional medical conditions (M = 1), and a significant medication change (*F* = 1). All participants who dropped out did so prior to TIME 1 or TIME 2.

### Descriptive data

Participants in the complete case analysis included 5 males and 5 females ranging in age from 18 – 64 years (average age = 34.6 ± 12.78SD). Participants who dropped out of the study, but for whom some data was available included 3 males and 4 females ranging in age from 18—68 (average age 40.57 ± 15.42). See Table 1 for demographic and clinical information.

**Table 1:** Demographics

**Demographics: Basic demographic and clinical characteristics for 10/10 complete cases**	
Male (%)	5 (50%)
Female (%)	5 (50%)
Mean age (range)	34.6 (18 – 64)
Hispanic	3 (30%)
African American	3 (30%)
Caucasian	4 (40%)
Focal or Multifocal Epilepsy (%)	3 (30%)
Generalized Epilepsy (%)	6 (60%)
Absence	1 (10%)
Antiepileptic drugs (%): baseline	
Phenytoin	1 (1%)
Clobazam	2 (20%)
Lacosamide	3 (30%)
Levetiracetam	4 (40%)
Carbamezepine	2 (20%)
Brivaracetam	2 (10%)
Zonasimide	1 (10%)
Lamotrigine	2 (20%)
Topiramate	1 (10%)
Valproate	1 (10%)
Vagal Nerve Stimulator	2 (20%)
**Demographics: Basic demographic and clinical characteristics for 7/11 dropouts**	
Male (%)	3 (43%)
Female (%)	4 (57%)
Mean age (range)	40.6 (18 – 68)
Hispanic	2 (29%)
African American	0 (0%)
Caucasian	5 (71%)
Focal or Multifocal Epilepsy (%)	1 (14%)
Generalized Epilepsy (%)	6 (86%)
Absence	5 (71%)
Antiepileptic drugs (%): baseline	
Phenytoin	0 (0%)
Clobazam	1 (14%)
Lacosamide	2 (29%)
Levetiracetam	2 (29%)
Carbamezepine	0 (0%)
Brivaracetam	0 (0%)
Zonasimide	2 (29%)
Lamotrigine	5 (71%)
Topiramate	0 (0%)
Valproate	0 (0%)
Vagal Nerve Stimulator	0 (0%)

Med data is for 6/7 participants

Basic demographic and clinical characteristics of the participants and dropouts are shown

### Outcome data

#### Main effect of time on the QOLIE-31-P

**Fig. 2:**
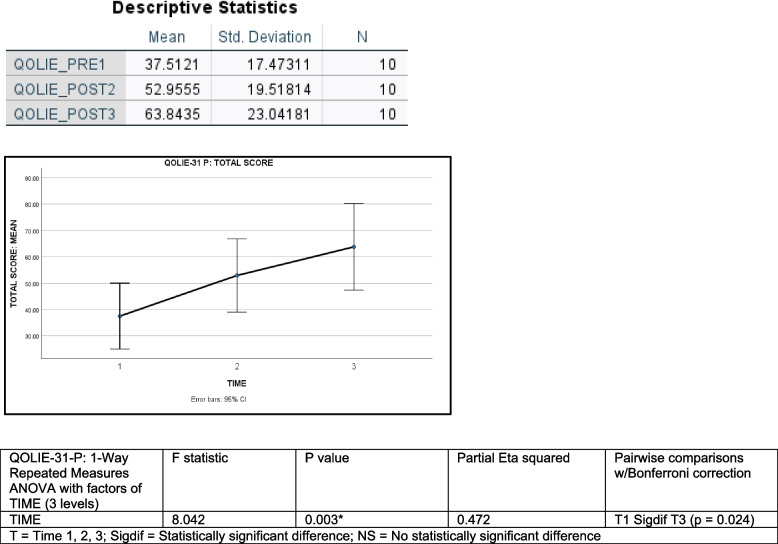
QOLIE-31-P: Quality Of Life in Epilepsy; Legend: Fig. 2 (top) shows the mean and standard deviation for the TOTAL SCORE on the QOLIE-31-P for each of the three time points during which data was collected. The chart below (middle) plots the QOLIE-31-P TOTAL SCORE means for each of the three time points, along with 95% Confidence Intervals. Fig. 2 (bottom) shows the results of a one-way repeated measures ANOVA on the QOLIE-31-P TOTAL SCORE with a factor of TIME (3 levels), as well as the results for Bonferroni-corrected pairwise comparisons

Descriptive and inferential statistics for the QOLIE-31-P are shown in Fig. 2.

The mean QOLIE-31-P TOTAL SCORE at enrollment (TIME 1) was 37.51 ± 17.47 SD, compared to 52.96 ± 19.52 SD at TIME 2, and 63.84 ± 23.04 SD at the end of the study (TIME 3), representing an average increase in QoL scores of 26.33 points over time. Higher scores were indicative of improved QoL. QOLIE-31-P SUBSCALE means also increased over time.

A one-way repeated measures ANOVA, with a factor of TIME (3 levels = TIME 1, 2, 3), found a significant main effect of TIME on the QOLIE-31-P TOTAL SCORE (2 df, *F* = 8.042, *p* = 0.003). This indicated that participants’ TOTAL SCORES increased over TIME. Partial Eta squared was 0.472, which was indicative of a large effect. Pairwise comparisons showed TIME 1 was significantly different from TIME 3 (*p* = 0.024), but not TIME 2 (*p* = 0.221). TIME 2 was also not significantly different from TIME 3 (*p* = 0.124). Therefore, the increase measured in TOTAL SCORE over TIME was largely due to the increase in scores between TIME 1 and TIME 3. This illustrated an improvement in QoL during the time-frame when participants were utilizing CBD.

#### Main effect of time on the HADS

**Fig. 3:**
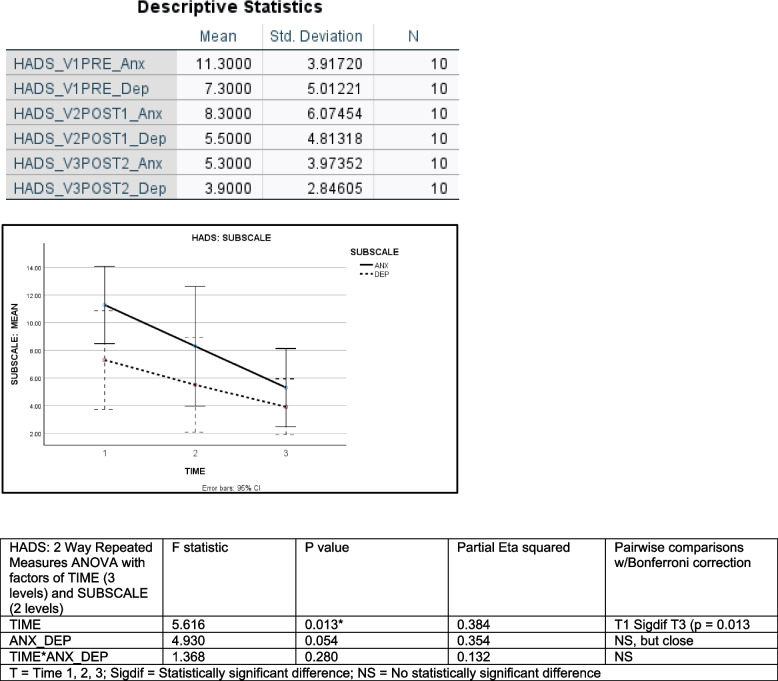
HADS: Hospital Anxiety and Depression Scale; Legend: Fig. 3 (top) shows the means and standard deviations for both the ANXIETY and DEPRESSION SUBSCALES of the HADS for TIMES 1, 2 and 3. The chart below (middle) plots the two sets of HADS SUBSCALE means for each of the three time points, along with 95% Confidence Intervals. Fig. 3 (bottom) shows the results of a two-way repeated measures ANOVA on data from the HADS with factors of TIME (3 levels) and SUBSCALE (2 levels), along with the results of Bonferroni-corrected pairwise comparisons

Descriptive and inferential statistics for the HADS are shown in Fig. 3.

The mean value for anxiety symptoms on the HADS ANXIETY SUBSCALE at TIME 1 was 11.3 ± 3.92 SD. The ANXIETY SUBSCALE scores decreased from 11.3 ± 3.92 SD at TIME 1 to 5.3 ± 3.97 SD at TIME 3, representing an improvement, on average, of 6 points. Levels of depression symptoms at TIME 1 were 7.3 ± 5.01SD. The HADS DEPRESSION subscale scores decreased from 7.3 ± 5.01 SD at TIME 1 to 3.9 ± 2.85 SD at TIME 3, representing an average improvement of 3.4 points. Mean ANXIETY and DEPRESSION scores both decreased over TIME.

HADS data was initially analyzed using a two-way repeated measures ANOVA, with factors of TIME (3 levels = TIME 1, 2, 3) and SUBSCALE (2 levels = anxiety, depression). A main effect of TIME was found (2 df, *F* = 5.616, *p* = 0.013). Partial Eta squared was 0.384. A main effect of SUBSCALE was almost significant (*p* = 0.054). Partial eta was 0.354. The interaction of TIME and SUBSCALE was not significant (*p* = 0.280). Pairwise comparisons showed TIME 1 was significantly different from TIME 3 (*p* = 0.013), but not TIME 2 (*p* = 0.461). Time 2 was not significantly different from TIME 3 (*p* = 0.407). These results indicated that one SUBSCALE was likely primarily responsible for the improvement observed in HADS scores between TIME 1 and TIME 3.

#### Main effect of time on the LAEP

**Fig. 4:**
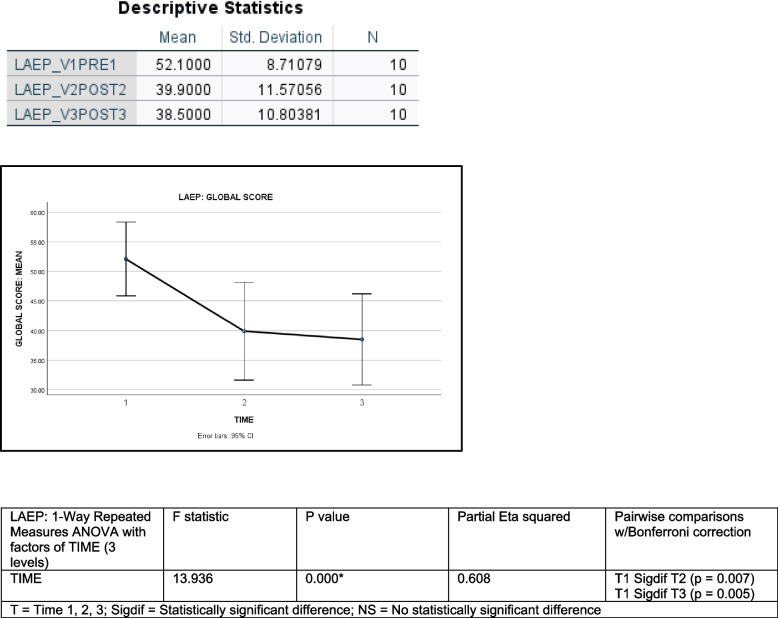
LAEP: Liverpool Adverse Events Profile; Legend: Fig. 4 (top) shows the mean and standard deviation for the LAEP GLOBAL SCORE for each of the three time points during which data was collected. The chart in middle shows the mean GLOBAL SCORES on the LAEP at TIMES 1, 2, and 3 along with 95% Confidence Intervals. Fig. 4 (bottom) shows the results of a one-way repeated measures ANOVA on GLOBAL SCORES from the LAEP with a factors of TIME (3 levels), along with the results of Bonferroni-corrected pairwise comparisons

Descriptive and inferential statistics for the LAEP are shown in Fig. 4.

LAEP GLOBAL SCORES were calculated by summing all responses to each LAEP item that indicated the symptom was “always” or “sometimes” a problem across participants. Some of the most frequently reported issues were: memory problems, difficulty concentrating, and nervousness/agitation. The mean GLOBAL SCORES for the LAEP decreased from 52.1 ± 8.71 SD at TIME 1 to 38.5 ± 10.80 SD at TIME 3, indicative of an average improvement of 13.6 points.

LAEP GLOBAL SCORES were subsequently analyzed using a one-way repeated measures ANOVA, with a factor of TIME (3 levels). A highly significant main effect of TIME was found (2df, *F* = 13.936, *p* = 0.000). Partial Eta was 0.608, which indicated a very large effect. Estimated marginal means confirmed a decrease in global LAEP scores over TIME. Pairwise comparisons showed TIME 1 was significantly different from TIME 2 (*p* = 0.007) and TIME 3 (*p* = 0.005). TIME 2 did not differ from TIME 3 (*p* = 1.000). This means that there was a significant decrease in adverse medication effects within the first 2.5 months of CBD use that continued over the course of the study, albeit at a slower rate.

#### Other analyses

Inferential statistics for the QOLIE-31-P and the HADS SUBSCALES are shown in Table 2.

**Table 2:** Post-hoc analyses

	F statistic	*P* value	Partial Eta squared	Pairwise comparisons w/Bonferroni correction
**QOLIE-31-P: 1-Way Repeated Measures ANOVA with factor of TIME (3 levels)**				
Subscale A (Energy)	3.851	0.041^*^	0.300	NS
Subscale B (Mood)	4.650	0.024^*^	0.341	NS
Subscale C (Daily Activities)	4.459	0.027^*^	0.331	T1 Sigdif T3 (*p* = 0.032)
Subscale D (Cognition)	6.046	0.010^*^	0.402	T1 Sigdif T3 (*p* = 0.032)
Subscale E (Medication Effects; *n* = 8)	0.992	0.396	0.124	N.A.
Subscale F (Seizure Worry)	8.070	0.003 (G-G = 0.014^*^)	0.473	T1 Sigdif T3 (*p* = 0.041)
Subscale G (Overall Quality of Life)	5.328	0.015^*^	0.372	NS
Subscale (Distress)	8.687	0.002 (G-G = 0.010^*^)	0.491	T1 Sigdif T3 (*p* = 0.036
**HADS: 1-Way Repeated Measures ANOVAS with factors of TIME (3 levels)**				
TIME (ANXIETY)	5.718	0.012^*^	0.388	T1 Sigdif T3 (*p* = 0.021
TIME (DEPRESSION)	2.859	0.083	0.241	T1 Sigdif T3 (*p* = 0.013

(top) shows the results of multiple one-way repeated measures ANOVAS on SUBSCALE data from the QOLIE-31-P with a factor of TIME (3 levels), along with the results of Bonferroni-corrected pairwise comparisons. Table 22 (middle) shows the results of a one-way repeated measures ANOVA on data from the HADS ANXIETY SUBSCALE with a factor of TIME (3 levels), along with the results of Bonferroni-corrected pairwise comparisons. Table 22 (bottom) shows the results of a one-way repeated measures ANOVA on data from the HADS DEPRESSION SUBSCALE with a factor of TIME (3 levels), along with the results of Bonferroni-corrected pairwise comparisons

G-G = Greenhouse-Geisser correction for Mauchly’s Test of Sphericity violation

T = Time 1, 2, 3, *Sigdif* Statistically significant difference, *NS* No statistically significant difference

*p < 0.05

Post hoc one-way repeated measures ANOVAs showed a main effect of TIME on almost all of the QOLIE-31-P SUBSCALES, including: distress (2df, *F* = 8.687, *p* = 0.010), cognition (2df, *F* = 6.046, *p* = 0.010), seizure worry (2df, *F* = 8.070, *p* = 0.014), overall quality of life (2df, *F* = 5.328, *p* = 0.015), mood (2df, *F* = 4.65, *p* = 0.024), daily activities (2df, *F* = 4.459, *p* = 0.027), and energy (2df, *F* = 3.851, *p* = 0.041) (Fig. 3). Partial Eta’s on significant tests ranged from 0.300 to 0.491, and were indicative of large effects. Two subscales required Greenhouse–Geisser corrections due to sphericity violations (distress and seizure worry). No main effect of TIME was found for medication effects (2df, *F* = 0.992, *p* = 0.396). Pairwise comparisons showed TIME 1 was significantly different from TIME 3 for the following subscales: distress (*p* = 0.036), cognition (*p* = 0.032), seizure worry (*p* = 0.041), and daily activities (*p* = 0.032). No other post hoc tests were statistically significant. While many of the subscale scores increased over time, those that contributed most to the statistically significant difference in TOTAL SCORE found between TIME1 and TIME 3 were: distress, cognition, seizure worry, and daily activities.

HADS data was further analyzed using two separate one-way repeated measures ANOVAs in order to independently assess the effects of TIME on ANXIETY vs. DEPRESSION. A main effect of TIME on ANXIETY was found (2 df, *F* = 5.718, *p* = 0.012). Partial Eta squared was 0.388. Post hoc pairwise comparisons showed TIME 1 was significantly different from TIME 3 (*p* = 0.021) but not TIME 2 (*p* = 0.251). Time 2 was not significantly different from TIME 3 (*p* = 0.513). No main effect was found for TIME on DEPRESSION (2 df, *F* = 2.859, *p* = 0.083). Although estimated marginal means confirmed a decreasing trend in depression scores over time, this data indicated that ANXIETY decreased more over TIME than DEPRESSION.

The missing data analysis was performed using data from the 7/11 dropouts for which we had TIME 1 data as opposed to no data (Table 3). Two of the three variables were missing data, 41.2% of cases were missing data and 27.45% of values were missing. Pattern analyses indicated that data was potentially Missing Not At Random (MNAR) [24]. This information was used to inform our choice of the “montone” method of handling missing data.

**Table 3:** Missing data analyses: imputed data analysis

Multiple Imputation datasets: QOLIE-31-P, HADS, LAEP				
Imputation dataset ^#^	N	F statistic	*P* value	Partial ETA Squared
QOLIE-31-P: 1-Way Repeated Measures ANOVA with factor of TIME (3 levels): (Greenhouse-Geisser corrected for sphericity violation)				
Original complete case dataset	10	8.042	.003^*^	.472
1	17	8.850	<.001^*^	.356
2	17	5.087	.012 (.021)^*^	.241
3	17	13.858	<.001 (.001)^*^	.464
4	17	8.371	.001 (.003)^*^	.343
5	17	3.553	.04 (.058)	.182
HADS: 2-Way Repeated Measures ANOVA with factors of TIME (3 levels) and SUBSCALE (2 levels)				
Original complete case dataset	10	5.616	.013^*^	.384
1	17	7.972	.002^*^	.333
2	17	8.174	.001^*^	.338
3	17	2.048	.146	.113
4	17	3.010	.063	.158
5	17	6.755	.004^*^	.297
LAEP: 1-Way Repeated Measures ANOVA with factor of TIME (3 levels)				
Original complete case dataset	10	13.936	<.001^*^	.608
1	17	21.973	<.001^*^	.579
2	17	1.062	.357	.062
3	17	6.975	.003^*^	.304
4	17	13.647	<.001 (.001)^*^	.460
5	17	14.845	<.001^*^	.481

(top) shows the results of one-way repeated measures ANOVAS with a factor of TIME (3 levels) for five imputed datasets for the QOLIE-31-P, along with the original complete case results. Table 2 (middle) shows the results of two-way repeated measures ANOVA for the HADS with factors of TIME (3 levels) and SUBSCALE (2 levels) for five imputed datasets, along with the original complete case results. Table 2 (bottom) shows the results of one-way repeated measures ANOVA with a factor of TIME (3 levels) for five imputed datasets for the LAEP, along with the original complete case results

^*^ = *p*<0.05

^#^ = imputed dataset number

Results of repeated measures ANOVAs for imputed QOLIE-31-P data showed that 4/5 imputations had a significant main effect of TIME. P values for 4/5 imputations required Greenhouse–Geisser corrections due to sphericity violations, 3 out of 4 of the corrected *p* values remained statistically significant: (MI(1), *p* =  < .001, MI(2) *p* = .021, MI(3) *p* = .001, MI(4) *p* = .003, MI(5) *p* = .058, original data, *p* = .003). Results of repeated measures ANOVAs for imputed HADS data showed that 3/5 imputations had a significant main effect of TIME: (MI(1), *p* = .002, MI(2) *p* = .001, MI(3) *p* = .146, MI(4) *p* = .063, MI(5) *p* = .004, original data, *p* = .013). Results of repeated measures ANOVAs for imputed LAEP data showed that 4/5 imputations had a significant main effect of TIME. One of these *p* values required a Greenhouse–Geisser correction, the corrected p value remained statistically significant: (MI(1) *p* =  < .001, MI(2) *p* = .357, MI(3) *p* = .003, MI(4) *p* =  < .001, MI(5) *p* =  < .001, original data, *p* =  < .001).

### Urinalysis

Cannabinoids and metabolites found in participants’ urine included: THC, 11-OH-THC, THCCOOH, THCCOOH-Gluc, THC-Gluc, CBD, 6a-OH-CBD, 6b-OH-CBD, 7-OH-CBD, 7-CBD-COOH, CBD-Gluc. The only metabolites tested for and not found in any of the urine samples were: CBC, CBN, CBG, THCV, and CBDV. Here we report on results for the primary CBD and THC metabolites-only.

### The primary metabolites for CBD excretion detected in participants’ urine were CBDCOOH and CBD-Gluc

The median concentration of CBDCOOH at TIME 1 was 0.00 ng/mL; the mean was 3.11 ng/mL; the range was (0.00 – 24.84 ng/mL). The median concentration of CBDCOOH at TIME 2 was 1.39 ng/mL; the mean was 22.09 ng/mL; the range was (0.00 – 157.18 ng/mL). The median concentration of CBDCOOH at TIME 3 was 4.184 ng/mL; the mean was 24.76 ng/mL; the range was (0.00 -126.82 ng/mL).

The median CBD-Gluc concentrations detected in participants' urine at TIME 1 was 0.00 ng/mL; the mean was 7.25 ng/mL; the range was (0.00 – 32.52 ng/mL). The median CBD-Gluc concentration at TIME 2 was 162.52 ng/mL; the mean was 180.20 ng/mL; the range was (24.192 – 473.70 ng/mL). The median CBD-Gluc concentration at TIME 3 was 77.16 ng/mL; the mean was 173.55 ng/mL; the range was (7.75 – 547.27 ng/mL).

The primary metabolites for THC excretion detected in participant’s urine were THCCOOH and THCCOOH-Gluc. The median THCCOOH concentration detected at TIME 1 was 0.25 ng/ml; the mean was 2.20 ng/mL; the range was (0.00 – 9.11 ng/mL). The median THCCOOH concentration detected at TIME 2 was 0.00 ng/mL; the mean was 12.70 ng/mL; the range was (0.00 – 106.54 ng/mL). The median THCCOOH concentration detected at TIME 3 was 0.94 ng/mL; the mean was 26.38 ng/mL; the range was (0.00 – 248.75 ng/mL).

The median THCCOOH-Gluc concentration detected at TIME 1 was 0.00 ng/mL; the mean was 153.36 ng/mL; the range was (0 – 1196.96 ng/mL). The median THCCOOH-Gluc concentration detected at TIME 2 was 18.07 ng/mL; the mean was 635.46 ng/mL; the range was (0 – 5478.00 ng/mL). The median THCCOOH-gluc concentration detected at TIME 3 was 9.766 ng/ml; the mean was 1309.71 ng/mL; the range was (0 -12529.83 ng/mL).

Levels of metabolites varied widely from below the lower limits of quantification (LLOQ) to above the upper limits of quantification (ULOQ) and differed across participants for different metabolites. The ULOQ was exceeded in 9/30 samples across five different people. Five of thirty possible urine samples were missing (3) or not tested with the second assay (2). All ten participants reported no cannabinoid use in the last 30 days. However, five of eight participants tested were positive for cannabinoids/metabolites at TIME 1.

## Discussion

### Key results

The results of this prospective, observational, longitudinal quasi-experimental time-series study on artisanal CBD use revealed that CBD use is associated with a statistically significant increase in QoL over time. These findings support our primary hypothesis that chronic adjunctive use of artisanal CBD was beneficial for adults with TRE. Critically, these results are consistent with previous studies showing improved QoL associated with the use of either Epidiolex or artisanal CBD in adults and children with TRE [25–27]. Although QoL is increasingly recognized as an important measure of treatment outcome [26, 28], determining its clinical significance still represents a challenge. While the present study used the QOLIE–31 P, the amount of change found to be clinically relevant for the QOLIE-31 (on which the QOLIE-31-P is based) was an 11.8 point change [29]. The mean increase in QoL scores from TIME 1 to TIME 3 was 26.33 points. This suggests that the improvement observed in QoL, associated with the adjunctive use of CBD, was not only statistically significant, but also clinically relevant.

While a statistically significant and clinically relevant level of improvement in the QOLIE-31-P TOTAL SCORE was found between TIME 1 and TIME 3, a clinically relevant level of change = 15.45 points was also found between TIME 1 and TIME 2 [29]. In addition, the level of change between TIME 2 and TIME 3 was = 10.88 points, which was just below clinical relevance. This suggests that the addition of CBD to an existing treatment regime had a clinically relevant effect on QoL after approximately two and one-half months of treatment, and that QoL improvement was sustained and reached a statistically significant level with an additional two and one-half months of CBD treatment. In addition, post-hoc analyses of the QOLIE-31-P SUBSCALES showed almost all improved over time and specific SUBSCALES showed statistically significant levels of improvement. Subscales with statistically significant levels of improvement included: distress, seizure worry, cognition, and daily activities. As there is an urgent need to improve QoL in people with TRE, these results provide promising insights into the therapeutic potential of CBD. Although this data suggests daily adjunctive use of artisanal CBD may improve QOL for people with TRE of mixed etiologies, more controlled research is needed to determine the optimal CBD product choice, phytocannabinoids ratio, dosage, and route of administration.

Supporting our secondary hypothesis, we found a significant improvement in mood and psychological well-being in the form of a statistically significant decrease in anxiety, and a decreasing trend in depression symptoms that was associated with CBD use. This finding was supported by a growing body of evidence from both human and animal research studies pointing to the anxiolytic and antidepressant potential of CBD [30–34].The majority of participants in this study exhibited the probable presence of a clinically significant level of anxiety on the HADS at TIME 1. This level steadily decreased over the course of the study to a level considered to be in the normal range at TIME 3 [17]. At TIME 2, the mean HADS score was still indicative of the presence of anxiety, albeit at a lower level. This suggests that adjunctive CBD use beyond TIME 2 may be associated with continued improvements in anxiety. Participants also presented with the possible presence of depression at enrollment. However, the level of depression was just barely above the threshold [17]. This was inconsistent with previous reports of depression being the most prevalent comorbid condition in TRE [35]. Although depression symptoms decreased over the course of the study to within a normal level, the decrease in depressive symptoms did not reach statistical significance.

Evidence from studies examining CBD mechanisms of action further supports our findings of decreased anxiety and depression associated with CBD use. While CBD has a relatively low affinity for the main CB1 and CB2 cannabinoid receptors, it has a good affinity for serotonin 5HT-1a receptors, where it acts as a receptor agonist [36, 37]. The 5HT-1a receptor is an established anxiolytic target [30]. Because anxiety and depression are considered major problems for people with TRE [38], decreasing symptoms of these conditions was considered a meaningful therapeutic outcome. Additionally, it is possible that some of the improvement we found in QoL may be attributed to the effect CBD had on anxiety and depression, as previous studies have found QoL was predicted by mood [26, 39]. Decreasing anxiety, for example, could reasonably decrease distress and seizure worry, leading to improved cognition and more comfort with daily activities. Improving symptoms of anxiety and depression are important therapeutic outcomes in TRE. Symptoms of anxiety and depression should be more widely assessed and treated in TRE.

Interestingly, we also found a statistically significant decrease in reported adverse events over time that was associated with CBD use. This result was consistent with a previous study, which found improvement in an adverse events profile from enrollment to follow up with Epidiolex treatment [40]. It was also consistent with an observational study of artisanal CBD users where participants reported significantly better epilepsy medication tolerability compared to controls [27]. One possible explanation for these findings was that after adding CBD to their existing treatment regime, participants were better able to tolerate their regular medication side effects and CBD use did not cause any additional adverse events. Improving medication tolerance and decreasing side effects is an important goal of TRE treatment.

A substantial amount of data was missing in this study. According to Powney et al., 2014 “with larger percentages of missing data, there is greater potential for bias if non-completing participants are ignored in the analysis….studies with dropout rates > 15% are in need of missing data to be analyzed and addressed” [23]. Repeated measures ANOVA analyses of imputed data largely supported the results of the complete case analysis, with significant main effects of time found for 4/5 (80%) datasets imputed for the QOLIE-31-P data, 3/5 (60%) datasets imputed for the HADS, and 4/5 (80%) datasets imputed for the LAEP, strengthening our confidence in our conclusion. It should be noted, however, that it is possible for MI to inject bias, especially with small datasets and limited numbers of variables [24]. In addition, if data are MNAR, MI may lead to additional bias; the monotone MI method was chosen to mitigate this potential. Why data was missing in this study is not entirely clear, one possibility is that people may not have been prepared to acquire CBD on their own, but there are many other possibilities.

The urinalysis performed in this study was exploratory and provided only a few snap shots of cannabinoid levels over the course of the study for each participant. However, the data confirmed that the majority of participants enrolled in this study added an amount of CBD to their treatment regime that was measurable in their urine at TIME 2 and TIME 3. Median levels of the primary CBD metabolites CBDCOOH and CBDgluc at TIME 1 were 0.00 ng/mL, indicating that the majority of participants tested had no CBD in their system at the beginning of the study. Median CBD/metabolites increased to levels well above the LLOQ at TIME 2 and TIME 3, indicating that the majority of participants had a measurable amount of CBD/metabolites in their urine at these times.

The median level of THC/metabolites at TIME 1 were very low but not zero. Median levels of the primary THC metabolites THCCOOH and THCCOOHgluc were 0.25 ng/mL and 0.0 ng/mL, respectively at TIME 1. Median THC/metabolite levels increased to levels above the LLOQ for TIMES 2 and 3, indicating the majority of participants had a measurable level of THC/metabolites in their system at these times. Results of the urinalysis are consistent with the majority of participants reporting using the whole spectrum product—Charlotte’s Web.

Although participants reported not having used any cannabinoids in the last 30 days, they were not required to be cannabis naïve, which likely affected the urinalysis results at TIME 1. Some participants had low but measureable levels of CBD/THC metabolites in their system at the start of the study. Importantly, though, the mean levels of metabolites at TIMES 2 and 3 were substantially larger than at TIME 1, and an increasing trend in the mean was apparent for all metabolites over time, confirming that participants added CBD to their treatment regime.

Because pharmacokinetic processes are dynamic, and may change over time, they may be affected by the frequency and magnitude of drug use [41]. How long cannabis stays in your system varies depending on many different factors including frequency of use, potency, consumption method, body composition, nature of drug test, etc. [42]. We highly recommend incorporating urine and/or blood testing of cannabinoid levels in future studies of chronic artisanal CBD use so that researchers can begin to define therapeutic cannabinoid metabolite levels.

### Strengths and limitations

This study had a number of strengths and weaknesses. One strength of this study was the use of a within subjects research design. This allowed participants to serve as their own controls and limited the number of participants required to reach statistical significance assuming a large effect. This study also had the benefit of being longitudinal, which allowed us to track sustained effects of CBD on participants’ behavior. A further strength was that, through urinalysis, we were able to confirm that participants had added CBD to their treatment regime.

One important limitation of this study was that the results for the complete case analysis were based on data from only 10 participants. Power analysis indicated the sample size required to achieve 85% power to detect an estimated large effect (d ≥ 0.8) at a significance criterion of α = 0.05, was 34, for within-subjects repeated measures ANOVA. Despite our best recruitment efforts, we were only able to enroll 21 participants and only ten participants completed the study. Therefore, the study was underpowered and thus the analyses could lead to biased conclusions, such as false positives. Although the longitudinal nature of the study was positive, the length of enrollment made it difficult for some participants to complete the study. This limitation was mitigated by analyzing missing data using MI.

Another limitation was that because this study was observational and not interventional, participants chose their own cannabinoid products and controlled their own dosing and drug administration. While most participants (60%) chose to use Charlotte’s Web Advanced Formula Hemp Oil Extract, their dosing varied, and some participants chose to use other products. Although this could potentially impact the generalization of this study, research shows that participants prefer to have control over their own treatment [43]. A sense of control is considered integral to a patient’s perception of their health and well-being. Presumably, participants in this study managed their CBD dosing in a manner that felt most therapeutic for them personally, and this was reflected in their improved perception of QoL, improved psychological well-being, and diminished experience of adverse effects.

Since this was not a RCT, this study lacked control for the placebo effect. The role of the placebo effect is particularly important to acknowledge as it is known to be particularly strong in epilepsy drug studies [44], and in studies of cannabinoid use in epilepsy [45, 46]. Moreover, widespread media coverage of CBD effectiveness at reducing seizures, together with the belief that natural products may be safer and more effective than traditional pharmaceuticals, could have led to selection bias. We cannot exclude the possibility that the placebo effect and selection bias may be partially responsible for the findings of this study. Although observational studies have some inherent limitations which constrain their ability to define causality, their strengths include that they reflect daily practice more closely than RCTs in terms of both the heterogeneous nature of the populations that are included in research and the treatment that is received [47].

In addition, a well-known psychological effect, called the Hawthorne effect, must also be acknowledged as a limitation of this study. This term, coined by Henry A Landsberger, refers to the effect of awareness of being part of a research study has on participants. The concern is that participants change their behavior due to this awareness. This was a long study with multiple points of contacts with researchers which potentially heightened participants’ awareness of being in the study.

A further limitation of this study was that participants were not required to be cannabis naïve. This likely affected the urinalysis results at TIME 1. Moreover, urinalysis was performed at the end of the study and was not used as an exclusion criteria. In general, real-time testing of CBD/metabolites was not readily available at the time the study was conducted, but is recommended.

### Interpretation

Our results suggest that use of widely available artisanal CBD formulations may be associated with an improvement in anxiety and depression symptoms, and QoL in adults with TRE of varying etiologies. Use of CBD may also be associated with a decrease in the experience of ASM-related side effects, improving medication tolerability.

### Generalizability

Despite the inability to assess causality, one strength of an observational study is that it may reflect real life practices better than randomized control trials which have much more rigid protocols. Therefore, this study may be generalizable to adult men and women age 18 – 64 years with TRE of varying etiologies who may not be eligible to use Epidiolex, and want to use artisanal CBD as an adjunctive treatment. However, as acknowledged in the limitations, it is possible, for example, that selection bias played a role in these results.

## Data Availability

The dataset used and analyzed for the current study is available from the corresponding author on reasonable request.

## Abbreviations

ASM: Antiseizure medication
Anandamide: N-arachidonoylethanolamine
ANOVA: Analysis of variance
CB1: Cannabinoid receptor type 1
CB2: Cannabinoid receptor type 2
df: Degrees of freedom
ECS: Endocannabinoid system
F: Fisher statistic
LAEP: Liverpool adverse events profile
LLOQ: Lower limits of quantification
mg: Milligram
mL: Milliliter
n.d.: No date
ng: Nanogram
p: Probability value
RCT: Randomized control trial
SD: Standard deviation
SUDEP: Sudden unexplained death in epilepsy
THC: Delta-9-tetrahydro cannabinol
TRE: Treatment resistant epilepsy
TRPV1: Transient receptor potential cation channel subfamily V member 1
PPARG: Peroxisome proliferator- activated receptor gamma
GPR55: G-protein coupled receptor 55
ULOQ: Upper limits of quantification
QoL: Quality of life
QOLIE-31-P: Quality of life in epilepsy-31-Patient weighted
2-AG: 2-arachidonoylglycerol
5-HT-1a: 5-hydroxytryptamine 1a receptor
5-HT-2a: 5-hydroxytryptamine 2a receptor
HADS: Hospital anxiety and depression scale
THC: Δ9-tetrahydrocannabinol
11OH-THC: 11-hydroxy-Δ9-tetrahydrocannabinol
THC-COOH: 11-nor-Δ9-tetrahydrocannabinol-9-carboxylic acid
THC-Gluc: 11-nor-Δ9-tetrahydrocannabinol-9-carboxylic acid glucuronide
CBD: Cannabidiol
6a-OH-CBD: 6a-hydroxy cannabidiol
6b-OH-CBD: 6b-hydroxy cannabidiol
7OH-CBD: 7-hydroxy cannabidiol
7-CBD-COOH: 7-cannabidiol-9-carboxylic acid
CBD-Gluc: Cannabidiol-9-carboxylic acid glucuronide
CBC: Cannabichromene
CBN: Cannabinol
CBG: Cannabigerol
CBDV: Cannabidivarin
THCV: Δ9-tetrahydrocannabivarin

## References

[1] N.I.o.N.D.a Stroke (2015). The epilepsies and seizures: hope and help through research.

[2] Sander JW (1993). Some aspects of prognosis in the epilepsies: a review. Epilepsia.

[3] Loscher W, Klein P (2021). The pharmacology and clinical efficacy of antiseizure medications: from bromide salts to cenobamate and beyond. CNS Drugs.

[4] Kwan P, Brodie MJ (2000). Early identification of refractory epilepsy. N Engl J Med.

[5] Kwan P (2010). Definition of drug resistant epilepsy: consensus proposal by the ad hoc Task Force of the ILAE Commission on Therapeutic Strategies. Epilepsia.

[6] Brodie MJ (2012). Patterns of treatment response in newly diagnosed epilepsy. Neurology.

[7] Devinsky O, Penry JK (1993). Quality of life in epilepsy: the clinician's view. Epilepsia.

[8] Suurmeijer TP, Reuvekamp MF, Aldenkamp BP (2001). Social functioning, psychological functioning, and quality of life in epilepsy. Epilepsia.

[9] Szaflarski M (2006). Quality of life in medication-resistant epilepsy: the effects of patient's age, age at seizure onset, and disease duration. Epilepsy Behav.

[10] Uijl SG (2006). A cross-sectional study of subjective complaints in patients with epilepsy who seem to be well-controlled with anti-epileptic drugs. Seizure.

[11] Carpay JA, Aldenkamp AP, van Donselaar CA (2005). Complaints associated with the use of antiepileptic drugs: results from a community-based study. Seizure.

[12] Loring DW, Marino S, Meador KJ (2007). Neuropsychological and behavioral effects of antiepilepsy drugs. Neuropsychol Rev.

[13] Perucca P, Gilliam FG (2012). Adverse effects of antiepileptic drugs. Lancet Neurol.

[14] Cramer JA, Van Hammée G, N.S. Group (2003). Maintenance of improvement in health-related quality of life during long-term treatment with levetiracetam. Epilepsy Behav.

[15] Borghs S, de la Loge C, Cramer JA (2012). Defining minimally important change in QOLIE-31 scores: estimates from three placebo-controlled lacosamide trials in patients with partial-onset seizures. Epilepsy Behav.

[16] Cramer JA (1998). Development and cross-cultural translations of a 31-item quality of life in epilepsy inventory. Epilepsia.

[17] Zigmond AS, Snaith RP (1983). The hospital anxiety and depression scale. Acta Psychiatr Scand.

[18] Baker GA (1993). The initial development of a health-related quality of life model as an outcome measure in epilepsy. Epilepsy Res.

[19] Baker GA (1994). Development of a novel scale to assess life fulfillment as part of the further refinement of a quality-of-life model for epilepsy. Epilepsia.

[20] Baker GA (1995). Health-related quality-of-life issues: optimizing patient outcomes. Neurology.

[21] Gaston TE (2017). Interactions between cannabidiol and commonly used antiepileptic drugs. Epilepsia.

[22] Andersson M (2016). Simultaneous quantification of 11 cannabinoids and metabolites in human urine by liquid chromatography tandem mass spectrometry using WAX-S tips. Anal Bioanal Chem.

[23] Powney M (2014). A review of the handling of missing longitudinal outcome data in clinical trials. Trials.

[24] Sterne JA (2009). Multiple imputation for missing data in epidemiological and clinical research: potential and pitfalls. BMJ.

[25] Stockings E (2018). Evidence for cannabis and cannabinoids for epilepsy: a systematic review of controlled and observational evidence. J Neurol Neurosurg Psychiatry.

[26] Gaston TE (2021). Long-term safety and efficacy of highly purified cannabidiol for treatment refractory epilepsy. Epilepsy Behav.

[27] Strickland JC (2021). Cross-sectional and longitudinal evaluation of cannabidiol (CBD) product use and health among people with epilepsy. Epilepsy Behav.

[28] Miguel RS (2008). Measuring health-related quality of life in drug clinical trials: is it given due importance?. Pharm World Sci.

[29] Wiebe S (2002). Clinically important change in quality of life in epilepsy. J Neurol Neurosurg Psychiatry.

[30] Blessing EM (2015). Cannabidiol as a potential treatment for anxiety disorders. Neurotherapeutics.

[31] Zuardi AW (2013). Human experimental anxiety: actual public speaking induces more intense physiological responses than simulated public speaking. Braz J Psychiatry.

[32] Shannon S (2019). Cannabidiol in anxiety and sleep: a large case series. Perm J.

[33] Crippa JA (2018). Translational Investigation of the Therapeutic Potential of Cannabidiol (CBD): Toward a New Age. Front Immunol.

[34] Garcia-Gutierrez MS (2020). Cannabidiol: A potential new alternative for the treatment of anxiety, depression, and psychotic disorders. Biomolecules.

[35] Fiest KM (2013). Depression in epilepsy: a systematic review and meta-analysis. Neurology.

[36] Pertwee R (2008). The diverse CB1 and CB2 receptor pharmacology of three plant cannabinoids: Δ9-tetrahydrocannabinol, cannabidiol and Δ9-tetrahydrocannabivarin. Br J Pharmacol.

[37] Gaston TE, Friedman D (2017). Pharmacology of cannabinoids in the treatment of epilepsy. Epilepsy Behav.

[38] Mensah SA (2007). A community study of the presence of anxiety disorder in people with epilepsy. Epilepsy Behav.

[39] Szaflarski JP (2018). Long-term safety and treatment effects of cannabidiol in children and adults with treatment-resistant epilepsies: Expanded access program results. Epilepsia.

[40] Gaston T (2019). Quality of life in adults enrolled in an open-label study of cannabidiol (CBD) for treatment-resistant epilepsy. Epilepsy Behav.

[41] Lowe RH (2009). Extended urinary Δ9-tetrahydrocannabinol excretion in chronic cannabis users precludes use as a biomarker of new drug exposure. Drug Alcohol Depend.

[42] Huestis MA (2020). Free and glucuronide urine cannabinoids after controlled smoked, vaporized and oral cannabis administration in frequent and occasional cannabis users. J Anal Toxicol.

[43] Gloss D, Vickrey B (2014). Cannabinoids for epilepsy. Cochrane Database Syst Rev.

[44] Goldenholz DM, Goldenholz SR (2016). Response to placebo in clinical epilepsy trials—old ideas and new insights. Epilepsy Res.

[45] Knupp KG (2019). Prospective evaluation of oral cannabis extracts in children with epilepsy. Seizure.

[46] Press CA, Knupp KG, Chapman KE (2015). Parental reporting of response to oral cannabis extracts for treatment of refractory epilepsy. Epilepsy Behav.

[47] Yang W (2010). Observational studies: going beyond the boundaries of randomized controlled trials. Diabetes Res Clin Pract.

